# Sputtering yield for metal halide perovskite devices patterning

**DOI:** 10.1080/14686996.2026.2637353

**Published:** 2026-03-02

**Authors:** Erfu Wu, Sergey Tsarev, Xuqi Liu, Daria Proniakova, Sergii Yakunin, Maksym V. Kovalenko, Ivan Shorubalko

**Affiliations:** a Transport at Nanoscale Interfaces Laboratory, Empa - Swiss Federal Laboratories for Materials Science and Technology, Dübendorf, Switzerland; b Department of Chemistry and Applied Biosciences, Laboratory of Inorganic Chemistry, ETH Zürich, Zürich, Switzerland; c Laboratory for Thin Films and Photovoltaics, Empa - Swiss Federal Laboratories for Materials Science and Technology, Dübendorf, Switzerland

**Keywords:** Argon ion milling, perovskite, sputtering yield, sputtering rate, dry etching

## Abstract

Metal halide perovskites (MHPs) are emerging semiconductors with unique optoelectronic properties promising for highly rewardable applications. Water and polar solvents instability hinders the introduction of MHPs into CMOS technology infrastructure and is the main challenge for patterning and integration into electronic systems. Recently, dry etching in combination with standard lithography was demonstrated as a viable technology to address the problem. In this work, we investigate the dry etching of MHPs using argon (Ar) ion milling. Simulated etch rates using Ziegler’s model are validated with experimental measurements. Assuming a linear sum of elemental sputtering yields results in total sputtering yield values for complex MHPs (CsPbIBr_2_, CsPbBr_2_Cl) that agree well with experimental data. Interestingly, ignoring the organic part of the hybrid halide perovskite MAPbI_2_Br gives a valid estimation of the sputtering yield. At a typical processing ion energy of 700 eV, Ar milling achieves rates of approximately 1–2 nm/s across various perovskite compositions. Photodetectors (PDs) fabricated under optimized etching conditions retain typical photoresponse, demonstrating the device functionality can be preserved after etching.

## IMPACT STATEMENT

We experimentally validate the simulated sputtering yields of complex metal/hybrid halide perovskites. Controllable dry etching provides a reliable pathway for the fabrication of high-performance perovskite photodetectors and other devices.

## GRAPHICAL ABSTRACT

**Figure:**
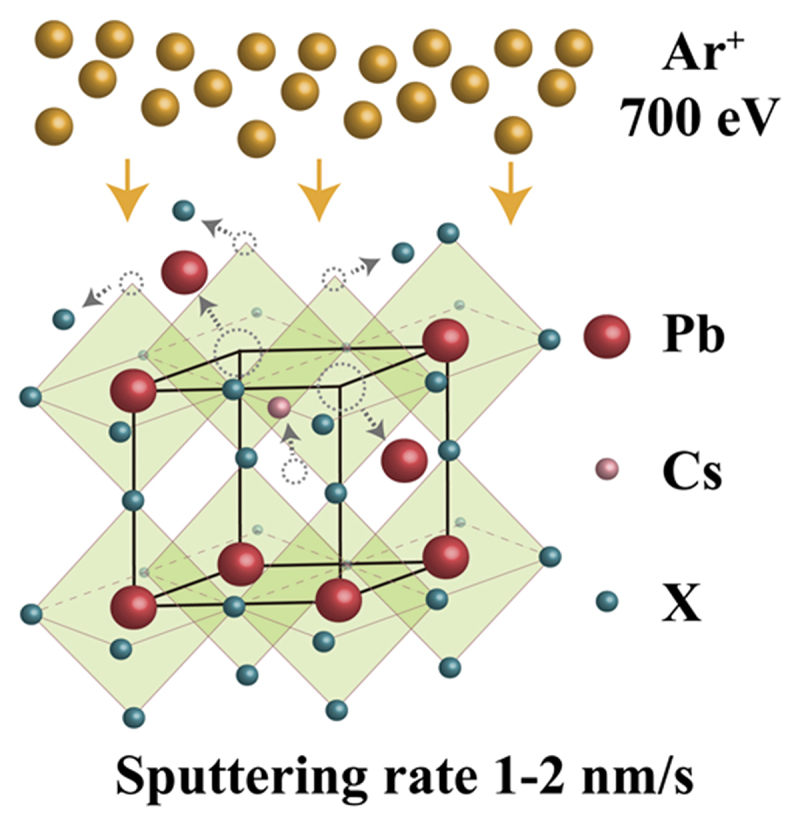

## Introduction

Since the pioneering demonstration of perovskite photovoltaic cells by Miyasaka in 2009 [1], metal halide perovskites (MHPs) have attracted tremendous attention owing to their superior optoelectronic properties, such as high absorption coefficients [2], sharp absorption edges [3], tunable bandgaps enabled by composition engineering [4], and solution processability [5]. Benefitting from these outstanding attributes, a wide range of MHP-based optoelectronic devices have been developed, including solar cells [6–9], photodetectors (PDs) [10–14], light-emitting diodes (LEDs) [15–18], radiation sensors [19–22] and memristors [23–26] in research community.

To enable the integration of MHPs into consumer electronics, reliable and scalable patterning technologies, such as lithography and etching, must be established. To date, three principle approaches for MHP patterning have been explored [27]: laser patterning, direct printing, and template-based patterning. Laser techniques can be employed either for material ablation [28–30] or for inducing localized crystallization from precursor films [31–33]. While submicrometer resolution has been demonstrated in patterning quantum dots through precise dose control, laser ablation or scribing of perovskite thin films typically results in feature sizes on the order of hundreds of micrometers and is therefore predominantly applied in large-area solar module fabrication. Direct printing approaches, including inkjet printing [34–36] and electrohydrodynamic printing [37–39], have also shown promise for patterning MHPs. However, these techniques generally rely on complex ink formulations, and achieving uniform film thickness and compositional homogeneity over device-relevant areas remains challenging. Template-based patterning employs pre-defined molds fabricated by micro-/nanoscale lithography [40–42] or millimeter-scale shadow masks [11]. In principle, nanometer-scale shadow masking is possible; however, its practical implementation has not yet been demonstrated, largely due to the complexity of fabrication and alignment. Following photoresist patterning – often enabled by spacer layers – etching of MHPs becomes essential to electrically isolate adjacent pixels and suppress crosstalk in electronic devices.

Despite its critical role, quantitative estimation of MHP etch rates remains insufficiently established. Due to their incompatibility with water and polar solvent, wet etching of MHPs is still being explored, while dry etching currently represents the more viable approach. Relevant reports have primarily focused on reactive ion etching (RIE) [43] and argon (Ar) ion sputtering [44]. However, these studies are limited to experimental observations without theoretical calculation. In addition, etch rates have been evaluated only for perovskite films rather than complete devices. For high-performing MHP-based electronic devices, charge transport layers (CTLs) are essential for efficient band alignment. In practical device architectures, such as PDs and LEDs, pixelation often requires etching of both the MHP absorbers and the CTLs, as the latter are typically more conductive and can cause electrical cross-talk in the absence of proper isolation [10].

In this study, we calculate the sputtering yield (***Y***) of MHPs with different bandgaps under Ar bombardment using Ziegler’s model with a linear summation of elemental contributions and validate through experimental measurements on MHP-based PDs. Despite the structural complexity and variability of MHPs, the presented study allows reliable dose estimation for etching of MHPs under Ar ions and establishes a straightforward approach for pixelating perovskite electronic devices.

## Calculation of MHPs sputtering yields

Accelerated ions have been widely employed in state-of-the-art complementary metal-oxide-semiconductor (CMOS) technology for decades, particularly in doping [45] and etching [46] processes. Among these techniques, Ar sputtering is one of the most versatile thanks to its chemical inertness and has been applied to the etching of novel materials like lithium niobate [47] and MHPs [44]. While theoretical models for estimating sputtering yields in single-element targets are well established, extending these calculations to compound materials is more complex and often imprecise, especially for hybrid systems where organic and inorganic components are intermixed.

**Figure 1.**
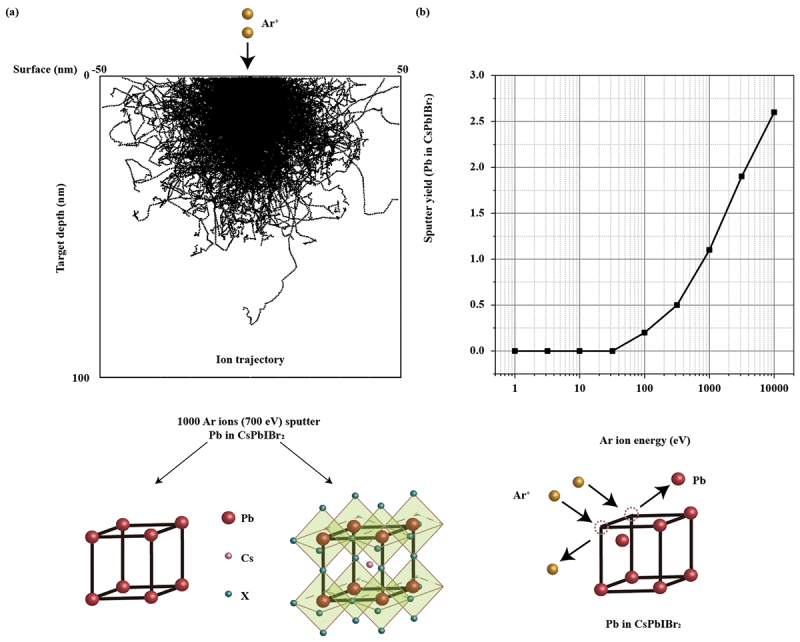
SRIM simulation of 1000 Ar ions bombarding Pb in CsPbIBr_2_. The mass density of Pb is adjusted according to the molar mass ratio in the complex perovskite CsPbIBr_2_. The effective mass density is calculated to be 1.55 g/cm^3^, considerably lower than that of pure Pb (~11.3 g/cm^3^). (a) The trajectories of Ar ions in the target. The incident Ar ions have an energy of 700 eV, which is the value used in the etching experiment. This is presented as an example in simulating the bombardment process. The trajectories of Ar ions in simulating Cs, I and Br targets can be referred to SI Figure 1 in Supplementary Information. (b) The (integral) sputter yield is plotted as a function of the energy of incident Ar ions. More details about the differential and integral sputter yield as well as energy dependence on Pb atoms are presented in SI Figures 2 and 3.

In Ziegler’s range theory [48], the sputtering yield is determined by evaluating the distribution of interactions between incident ions and single-element targets. As energetic ions strike the surface and undergo a series of scattering events before coming to rest, their interactions are described as both binary elastic collisions with the screened nuclei of target atoms and as inelastic collisions with the target’s electron system. Based on this framework, Ziegler developed the open-access SRIM (the Stopping and Range of Ions in Matter) software, which enables simulation of ion stopping processes in various targets. An example of Ar ions bombarding Pb (in CsPbI_2_Br) target is presented in Figure 1.

In this study, we estimated the sputtering yield of perovskites by applying a linear summation of the yields of their constituent elements under Ar ion bombardment using SRIM 2013. For example, in the case of CsPbIBr_2_, an MHP composition used in PDs for green light detection, the sputtering yield was calculated at an incident Ar ion energy of 700 eV, corresponding to the conditions employed in our etching experiments. The mass density of each element was derived from the molar mass fractions within the perovskite, whose overall mass density was estimated from database – Materials Project. Using this information, the elemental sputtering yields were calculated as:

***Y(Cs)*** = 1.5

***Y(Pb)*** = 1.0

***Y(I)*** = 0.9

***Y(Br)*** = 1.2

The total sputtering yield was then obtained by weighing the elemental contributions according to their stoichiometric fractions:

$$Y\left(\mathrm{CsPbIB}r_{2}\right)=Y(\mathrm{Cs})\times \frac{1}{5}+Y(\mathrm{Pb})\times \frac{1}{5}+Y(I)\times \frac{1}{5}+Y(\mathrm{Br})\times \frac{2}{5}=\;1.2$$

With the sputtering yield determined, the sputtering rate (***R***_***s***_) can be derived by considering the ion flux (***J***) and the atomic volume (***V***_***atom***_).

(1) $$\begin{array}{c} R_{s}\left(\mathrm{cm}/s\right)=Y\times J\times V_{\mathrm{atom}} \end{array}$$

The ion flux is defined as the product of ion velocity and ion dose, estimated as 6 × 10^4^ cm/s and 10^10^ ions/cm^3^, respectively, based on ion energy, mass, and literature values [49]. The atomic volume was calculated from the ratio of molar mass to the product of mass density and Avogadro’s number (***N***_***A***_).

(2) $$\begin{array}{c} V_{\mathrm{atom}}=\frac{\mathrm{Molarmass}}{\mathrm{Massdensity}\;\times \;N_{A}} \end{array}$$

Finally, the sputtering rate was then obtained as the product of sputtering yield, ion flux and atomic volume, expressed in nm/s. A summary of the estimated sputtering rates and relevant parameters is provided in Table 1. It shows that the sputtering rate of MAPbI_2_Br is approximately two times slower than that of the other two MHPs. To validate these calculated sputtering rates, perovskite PDs were fabricated using a lithographic process followed by dry etching.

**Table 1.** Estimated sputtering rates of perovskites to Ar ions with relevant parameters. The respective mass density is estimated from the online database *Materials Project*.

	*MAPbI*_*2*_*Br*	*CsPbIBr*_*2*_	*CsPbBr*_*2*_*Cl*
*Sputtering Yield (atoms/ion)*	*0.4*	*1.2*	*1.1*
*Molar Mass (g/mol)*	*573*	*627*	*535*
*Mass Density (g/cm*^*3*^)	*~4*	*~4.7*	*~4.5*
*V*_*atom*_ *(cm*^*3*^*/atom)*	*2.4 × 10*^*-22*^	*2.2 × 10*^*-22*^	*2 × 10*^*-22*^
*Sputtering Rate (nm/s)*	*0.6*	*1.6*	*1.3*

## Experimental sputtering rates of MHPs PDs

**Figure 2.**
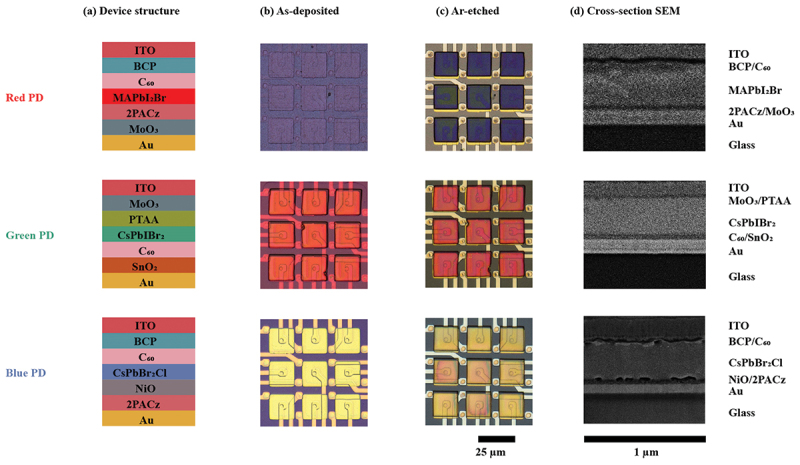
Ar ion sputtering of perovskite PDs. (a) Device structures of perovskite PDs for red, green and blue light detection. (b) Optical microscope images of as-deposited perovskite PDs. (c) Optical microscope images of etched perovskite PDs by Ar ions. Scale bar: 25 µm, Ref. [10]. (d) Cross-sectional SEM images of deposited perovskite PDs. Scale bar: 1 µm.

**Figure 3.**
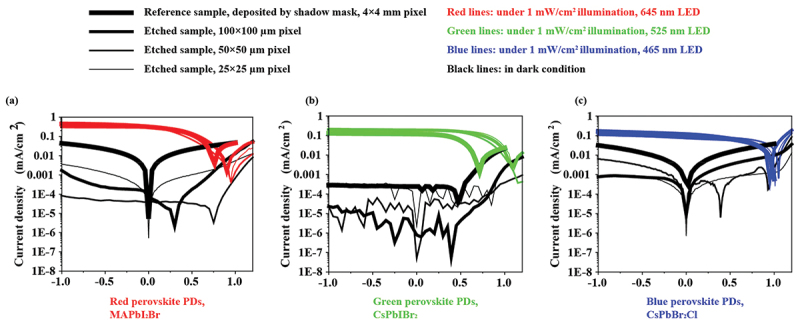
Typical J-V curves of dry etched perovskite PDs together with reference samples. (a) Red perovskite (MAPbI_2_Br) PD. (b) Green perovskite (CsPbIBr_2_) PD. (c). Blue perovskite (CsPbBr_2_Cl) PD. After Ar ion etching, PDs with varying pixel sizes retain the scalable photocurrent for typical MHP PDs. The resulting photocurrents are comparable to those of the reference devices, with a bit higher values observed for the red and green PDs.

The etching process was performed in a RIE tool (RIE 80, Oxford Instruments (UK)) using Ar as the sole source gas. Prior to etching, a lithographic process was carried out to pattern the photoresist. Details of the fabrication process can be referred to a previous literature [10]. The device structure along with cross-sectional SEM images and optical microscope images of the perovskite PDs after deposition and dry etching are presented in Figure 2. The deposition protocols for each perovskite PD are provided in the Supplementary Information. Following photolithography, pulsed Ar etching was employed to mitigate local heating of the samples. Each cycle consisted of 10 s of Ar etching followed by 60 s of cooling (no etching). During etching, a 400 W radio frequency (RF) power was applied, resulting in a sheath voltage of approximately 700 V (DC) with an Ar gas flow of 100 sccm. Complete etching was achieved after 600 s, 450 s, and 480 s of effective etching time for red, green, and blue perovskite PDs, respectively. This corresponds to 60, 45, and 48 cycles, with a total process duration of roughly 1 h. The complete etching of the perovskite PD was verified through optical inspection, where the strong contrast between perovskite absorber and the Au electrode is clearly visible under the optical microscope (Figure 2c). After dry etching, an AlO_x_ layer was deposited by atomic layer deposition (ALD) to encapsulate the etched sidewalls, and a contact window was subsequently fabricated by lithography followed by Ar etching. The PDs were completed by depositing ITO top electrode to bridge the source and drain electrodes. Typical J-V curves are presented in Figure 3, where the dry etched perovskite PDs exhibit photocurrent densities of approximately 0.3, 0.2 and 0.1 mA/cm^2^ for red, green and blue PDs respectively at 0 V under 1 mW/cm^2^ illumination. Other optoelectronic characterizations, including dark current density, spectral response, and temporal response, are reported in a previous study [10].

## Discussion

Table 1 summarizes the estimated sputtering rates of the three perovskites, showing that MAPbI_2_Br exhibits a considerably lower value than the other two compositions. This result appears counterintuitive, as the measured effective etching time for MAPbI_2_Br-based PDs (~600 s) is longer than that of the other devices (<500 s). The actual sputtering time of the perovskite absorber and CTLs was determined by subtracting the sputtering time required for the ITO top electrode from that of the full PD structure. This approach accounts for the degradation of perovskite films upon exposure to ambient air after removal from the glovebox, which can result in inaccurate material parameters for simulation. In contrast, when the perovskite layer is encapsulated by the top ITO and CTLs, it is effectively shielded from moisture and preserves its original composition. The contribution of the CTLs was assumed to be short and nearly constant. The experimental etching times obtained from these measurements are summarized in Table 2. It presents both the estimated values and the measured etching times. The estimated etching times were obtained from the deposited perovskite layer thickness and the sputtering rates listed in Table 1, while the total etching times for each perovskite PD, including the ITO top electrode, were experimentally recorded. Assuming negligible delays from intermixing at layer interfaces, the effective etching times of the perovskite absorbers were determined to be approximately 420 s, 310 s, and 360 s for the three PDs, respectively. Comparison with the estimated values shows that the calculated sputtering rates are reasonably accurate for CsPbIBr_2_ and CsPbBr_2_Cl, especially when accounting for an additional 50–100 s required to etch the CTLs. In contrast, a significant discrepancy arises in the case of the MAPbI_2_Br PD: the measured etching time for the perovskite is only about half of the value predicted for the perovskite layer alone. This inconsistency is likely due to the presence of organic components in the hybrid perovskite, which renders the sputtering yield model invalid for this composition.

**Table 2.** The layers in perovskite PD and measured etching time.

	*MAPbI*_*2*_*Br*	*CsPbIBr*_*2*_	*CsPbBr*_*2*_*Cl*
*Deposited Perovskite Layer Thickness (nm)*	*450*	*350*	*400*
***Calculated Etching Time for Perovskite layer (s)***	***750***	***220***	***310***
*Measured Etching Time for Perovskite PD (s)*	*600*	*450*	*480*
*Measured Etching Time for ITO (s)*	*180*	*140*	*120*
***Measured Etching Time for Perovskite Layer and CTL (s)***	***420***	***310***	***360***

To bring the model into closer agreement with the experimental data, we propose neglecting the organic component of the hybrid perovskite. In this approach, the absorber is treated as PbI_2_Br instead of MAPbI_2_Br, and the sputtering rate is recalculated using the same linear summation method. The comparison is summarized in Table 3. It shows that neglecting the organic groups in the hybrid perovskite improves the accuracy of the sputtering rate estimation. After this correction, the measured etching time for the perovskite and CTLs agrees well with the estimated value for the perovskite layer plus an additional ~100 s required for CTL etching, consistent with the behaviors observed in the other two perovskite PDs.

**Table 3.** Comparison of the etching process with and without organic groups.

	*MAPbI*_*2*_*Br*	*PbI*_*2*_*Br*
*Molar Mass (g/mol)*	*573*	*541*
*Sputtering Yield (atoms/ion)*	*0.4*	*1.1*
*Sputtering Rate (nm/s)*	*0.6*	*1.4*
*Deposited Perovskite Layer Thickness (nm)*	*450*	*450*
***Estimated Etching Time for Perovskite layer (s)***	***750***	***320***
*Measured Etching Time for Perovskite PD (s)*	*600*	*600*
*Measured Etching Time for ITO (s)*	*180*	*180*
***Measured Etching Time for Perovskite layer and CTL (s)***	***420***	***420***

## Conclusion

In this work, we systematically investigated the dry etching behavior of MHPs using Ar ion milling and compared the experimentally measured sputtering rates with theoretical calculations based on Ziegler’s model and linear summation assumption. We find that the sputtering rates of inorganic MHPs, such as CsPbIBr_2_ and CsPbBr_2_Cl, exhibit good agreement with the simulated values, confirming the validity of the linear combination of elemental sputtering yields for complex perovskites. For hybrid perovskites, the deviation from theoretical predictions can be effectively mitigated by neglecting the organic component in the complex. At a typical processing ion energy of 700 eV, the Ar milling rate reaches approximately 1–2 nm/s across different compositions. Furthermore, PDs fabricated under optimized etching conditions maintain typical photoresponse properties, demonstrating that perovskite devices can endure dry etching processes and compatibility with CMOS fabrication technology.

## Method

Information on the materials used for deposition and the detailed deposition protocols of the perovskite PDs are provided in the Supplementary Information. The Ar ion etching simulations were performed using SRIM 2013 Pro. The damage type in the simulation was chosen to be Monolayer Collision Steps/Surface Sputtering. All targets were treated as solid materials and the compound correction factor was 1. Displacement energy, surface binding energy, and lattice binding energy were adopted directly from the software database.

The etching experiments were carried out using RIE 80 from Oxford Instrument (UK). Ar gas was supplied at a flow rate of 100 sccm, with a chamber pressure of 75 mTorr and an RF power of 400 W, resulting in DC bias of approximately 700 V. To mitigate thermal accumulation during Ar ion milling, the dry etching process was operated in a pulsed mode, consisting of 10 s etching followed by 60 s rest intervals. The total etching time reported corresponds to the sum of effective etching duration over all cycles.

## Supplementary Material

Supplemental Material

## Data Availability

The data that supports the findings of this study are available within the article.
