# Supplementary material for: Sputtering yield for metal halide perovskite devices patterning
Source: Sci Technol Adv Mater. 2026 Mar 2;27(1):2637353. doi: 10.1080/14686996.2026.2637353 (PMC12997472; doi:10.1080/14686996.2026.2637353)
Supplement: Supplemental Material [file TSTA_A_2637353_SM9612.pdf]

Supporting Information

Sputtering yield for metal halide perovskite devices patterning

Erfu Wu[a], Sergey Tsarev[b], Xuqi Liu[b], Daria Proniakova[b], Sergii Yakunin[b], Maksym V. Kovalenko[b]*, Ivan Shorubalko[a]*

[a] *Transport at Nanoscale Interfaces Laboratory, Empa, Dübendorf 8600, Switzerland;*

[b] *Department of Chemistry and Applied Biosciences, Institute of Inorganic Chemistry, ETH Zürich, Zürich 8093, Switzerland*

ivan.shorubalko@empa.ch;

mkkovalenko@ethz.ch

Perovskite PD deposition protocol

Red Perovskite PD

Device fabrication commenced with thermal evaporation of a 30 nm MoO_3 buffer layer. A self-assembled monolayer of 2PACz (1.2 mg/mL in ethanol) was subsequently deposited by spin coating to serve as the hole transport layer. The perovskite absorber layer, consisting of a 450 nm $MAPbI_2Br$ film, was co-evaporated from MABr and PbI_2 sources. Electron transport layers, including 40 nm C_{60} and 8 nm BCP, were then deposited sequentially by thermal evaporation. Finally, a 180 nm ITO top electrode was deposited by sputtering to complete the device stack.

Green Perovskite PD

The green device fabrication began with sputter deposition of a 40 nm SnO_2 layer, followed by thermal evaporation of a 5 nm C_{60} film, both serving as electron transport layers. The perovskite absorber, $CsPbIBr_2$, was co-evaporated from CsI and $PbBr_2$ precursors. A 40 nm PTAA film was then spin coated as the hole transport layer, followed by thermal evaporation of a 30 nm MoO_3 buffer. The device was finalized by sputtering a 140 nm ITO layer as the top electrode.

Blue Perovskite PD

For the blue device, a thin (sub-2 nm) 2PACz layer was first spin-coated, followed by deposition of a 40 nm NiO nanocrystal layer functioning as the hole-transport layers. The perovskite absorber, a 400 nm $CsPbBr_2Cl$ film, was co-evaporated using CsCl and $PbBr_2$ sources. Electron transport layers comprising C_{60} (20 nm), BCP (8 nm), and Mg (2 nm) were sequentially thermally evaporated; the thin Mg layer enhanced device stability and adhesion by oxidizing during subsequent ITO deposition. The stack was completed with sputtering of a 120 nm ITO top electrode.

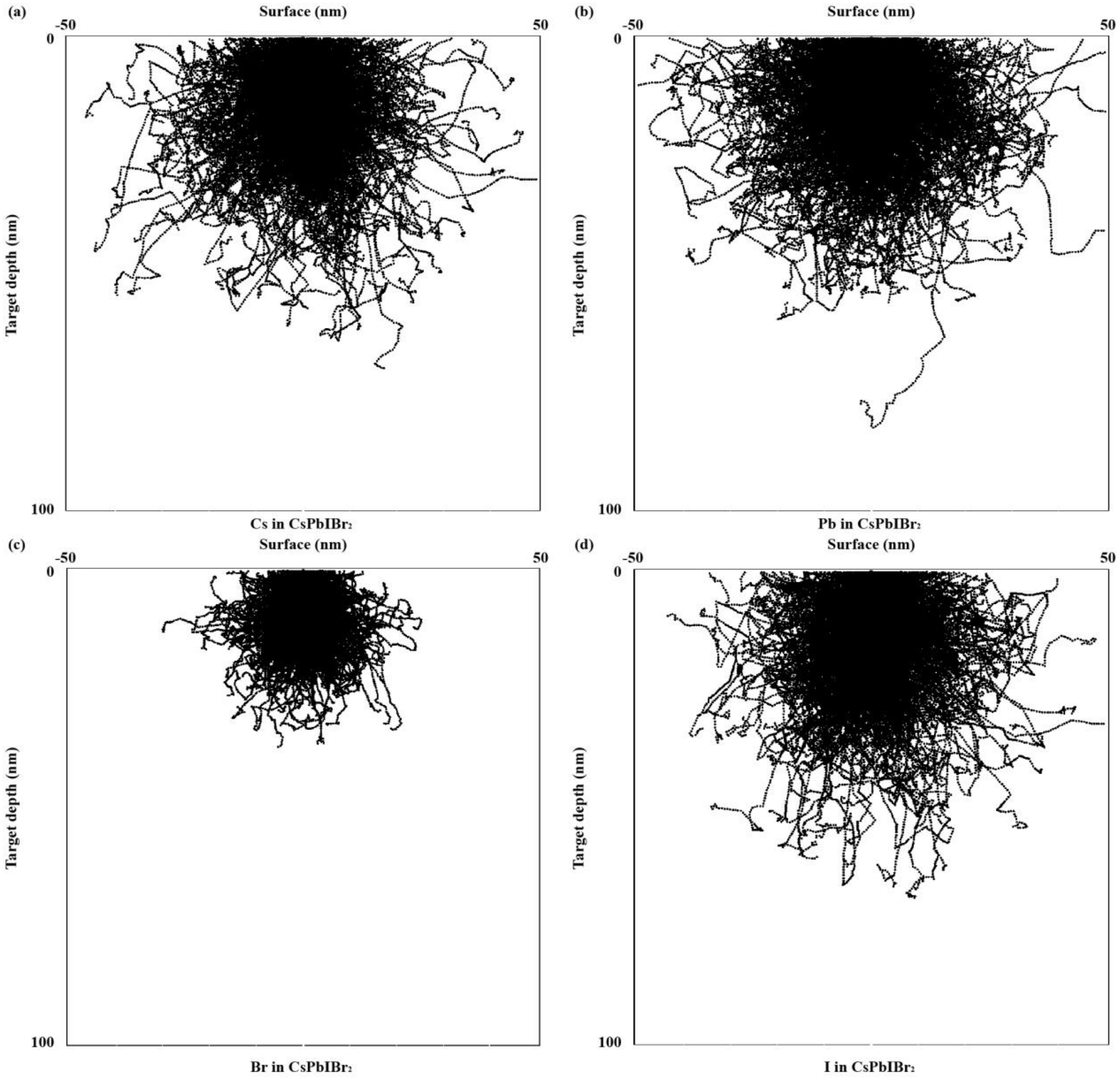

SI Figure 1. **Simulated Ar ion trajectories in elemental targets: (a)** Cs in $CsPbIBr_2$. **(b)** Pb in $CsPbIBr_2$. **(c)** Br in $CsPbIBr_2$. **(d)** I in $CsPbIBr_2$. Simulations were conducted with 1000 Ar ions at 700 eV under normal incidence (0°). Elemental mass densities were determined from their molar mass fractions. All binding and displacement energies were taken from the SRIM 2013 Pro database, with a target depth of 100 nm.

Next, the elemental sputter yield and its dependence on the ion incident angles are presented in **SI Figure 2**and **SI Figure 3**, using Pb in $CsPbIBr_2$ as a representative example.

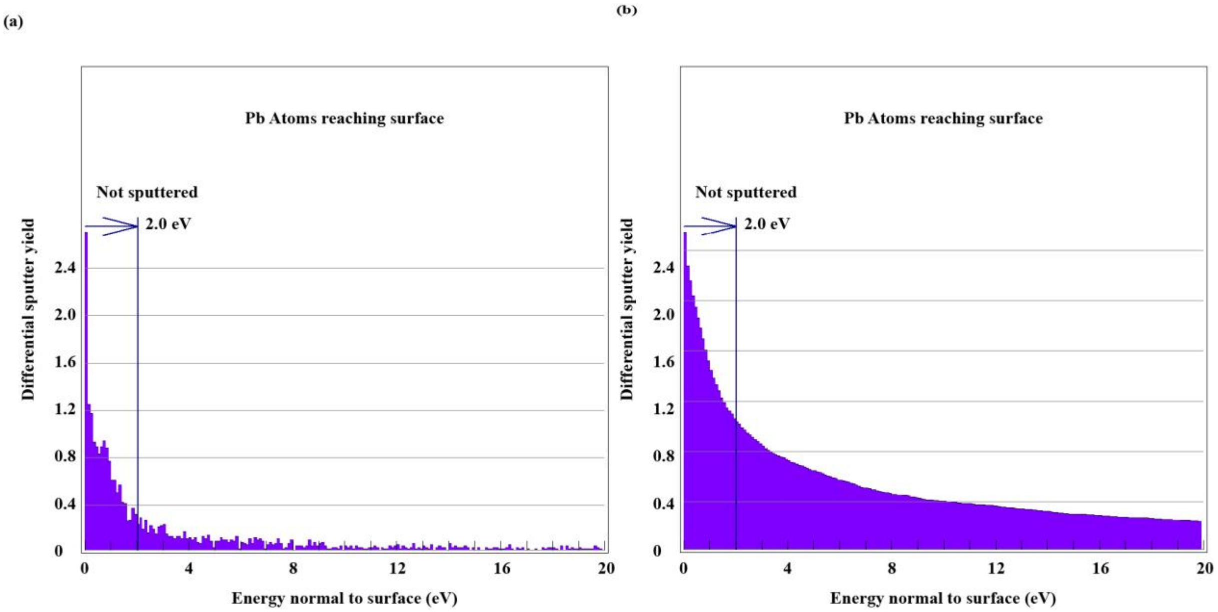

SI Figure 2. **Simulated sputter yield of Pb atoms in $CsPbIBr_2$. (a)** Differential and **(b)** integral sputter yields of Pb atoms. Following the collision cascade, Pb atoms exhibit a broad energy distribution; only atoms with a surface-normal energy exceeding the surface binding energy (2 eV) are able to escape from the target. Simulations were conducted with 1000 Ar ions at 700 eV under normal incidence (0°). Pb mass density was determined from its molar mass fractions. All binding and displacement energies were taken from the SRIM 2013 Pro database, with a target depth of 100 nm.

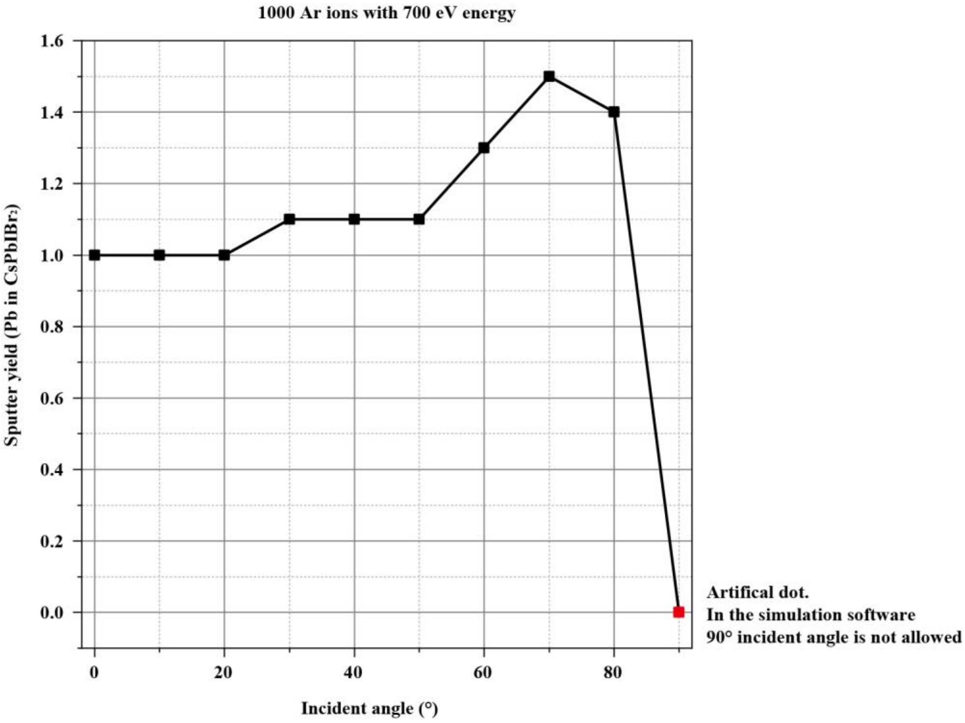

SI Figure 3. **Simulated sputter yield of Pb in $CsPbIBr_2$ as a function of Ar ion incident angle.** The sputter yield exhibits a maximum at incident angles between 60° and 80°, followed by a decrease at higher angles. The data point at 90° is shown for reference only, as this angle cannot be directly simulated in the software. Simulations were conducted with 1000 Ar ions at 700 eV under varying incident angles. Pb mass density was determined from its molar mass fractions. All binding and displacement energies were taken from the SRIM 2013 Pro database, with a target depth of 100 nm.
